# Proximal humerus fractures in the elderly work up, classifications and fracture biomechanics

**DOI:** 10.1007/s00068-025-02945-0

**Published:** 2025-10-16

**Authors:** Dario Kalacun, Radko Komadina, Drago Brilej

**Affiliations:** 1 General and Teaching hospital Celje, Celje, Slovenia; 2 Medical Faculty University Ljubljana, Ljubljana, Slovenia

**Keywords:** Proximal humerus fractures, Work up, Classifications, Fracture biomechanics

## Abstract

This article discusses fractures of the upper third of the humerus in elderly patients within the context of diagnostic evaluation, fracture classification, biomechanics, and fracture occurrence, including osteoporosis. The shoulder joint is composed of the proximal humerus and the glenoid of the scapula. Very important factors for fracture incidence and patterns are osteoporosis and distribution of bone tissue and bone density in the proximal humerus. Most fractures are caused by fall from standing hight. Parts of diagnostic work-up and management plans are clinical examination, different imaging modalities and classification sistems.

## Introduction

This article discusses fractures of the upper third of the humerus in elderly patients within the context of diagnostic evaluation, fracture classification, biomechanics, and fracture occurrence, including osteoporosis. These fractures are among the six most common osteoporosis-related fractures in the geriatric population, along with distal radius fractures, proximal femur fractures, vertebral fractures in the thoracic spine and thoracolumbar junction, as well as pelvic implosion-type fractures and periprosthetic fractures in individuals over 80 and 90 years of age.

These fractures typically occur in the epiphyseal and metaphyseal regions, where osteoporosis leads to a decrease in trabecular bone density. They are commonly caused by falls from a standing height and are classified as low-energy fractures. The diagnostic process includes a detailed clinical examination of the injured patient and imaging techniques that enable accurate fracture classification. Based on these steps, the appropriate treatment approach is determined, which is further discussed in other articles within this monograph.

## Applied anatomy

The shoulder joint is composed of the proximal humerus and the glenoid of the scapula, which is made of dense cortical bone. The glenoid labrum surrounds the periphery of the glenoid, increasing the depth of the shallow glenoid fossa by approximately 50%. Although the glenoid is congruent with the humeral head, its surface area is only 25–30% of the humeral head, providing minimal skeletal stability [1]. The tendon of the long head of the biceps muscle originates from the superior rim of the glenoid [2]. The joint is surrounded by a capsule reinforced by ligaments, which are relatively lax. The most important of these is the inferior glenohumeral ligament. Glenohumeral ligaments with coracohumeral ligament are passive stabilizers of the joint. The plane of the glenohumeral joint is approximately 30 degrees anterior to the coronal plane [3].

The proximal humerus consists of the head, two tubercles, and the shaft. The junction between the cartilaginous portion and the surrounding bone is termed the anatomic neck, while the region below the greater and lesser tubercles is called the surgical neck. The mean radius of curvature of the humeral head is 25 mm, and its size ranges from 40 to 50 mm. The head is slightly offset medially and posteriorly relative to the humeral shaft. In the coronal plane, the angle between the anatomic neck and the humeral shaft avenges 41° (range: 30–50°). In the axial plane, the anatomic neck of the humerus is angled at 17° of retroversion (range: 5° anteversion to 50° retroversion) relative to the transepicondylar axis of the distal humerus [4].

**Fig. 1:**
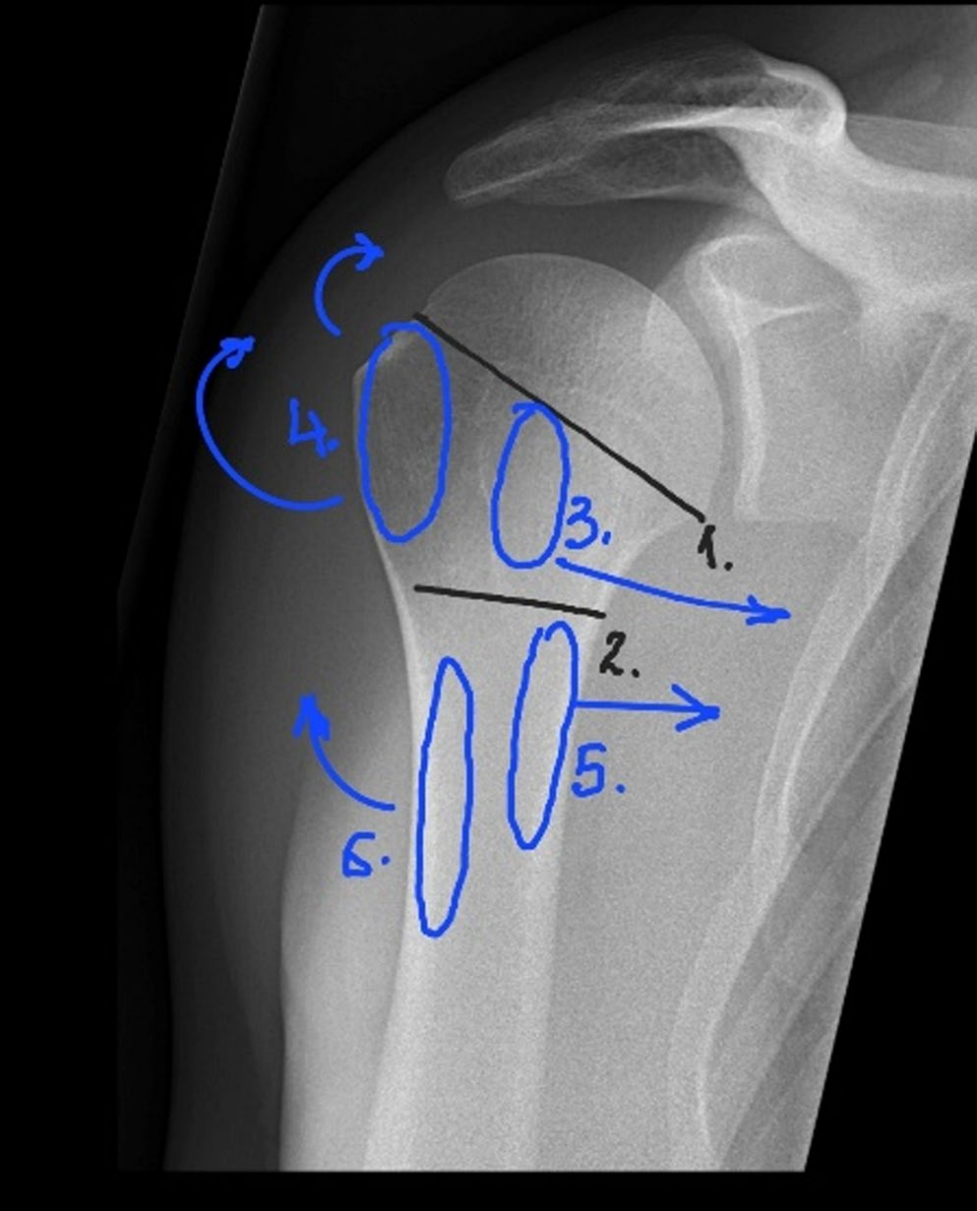
Proximal Humerus Anatomy (1. Anatomic Neck of the Humerus, 2. Surgical Neck of the Humerus, 3. Greater Tubercle – Insertion of the Supraspinatus, Infraspinatus and Teres Minor Muscle Tendons, 4. Lesser Tubercle – Insertion of the Infraspinatus Muscle Tendon, 5. Insertion of the Pectoralis muscle tendon, 6. Insertion of the Deltoid Muscle Tendon) [46]

The greater and lesser tubercles are separated anteriorly by the bicipital groove, which is occupied by the long tendon of the biceps brachii. The greater tubercle, located laterally, serves as the attachment site for the tendons of the supraspinatus (superiorly), infraspinatus (slightly posteriorly), and teres minor (posteriorly). The lesser tubercle, located anteriorly, serves as the attachment for the subscapularis tendon. The tendons and muscles of the rotator cuff are critical active stabilizers of the shoulder joint. The deltoid muscle attaches to the lateral aspect of the humeral diaphysis, while the pectoralis major inserts anteromedially, 5–6 cm distal to the apex of the humeral head [5] (Fig. 1).

**Fig. 2:**
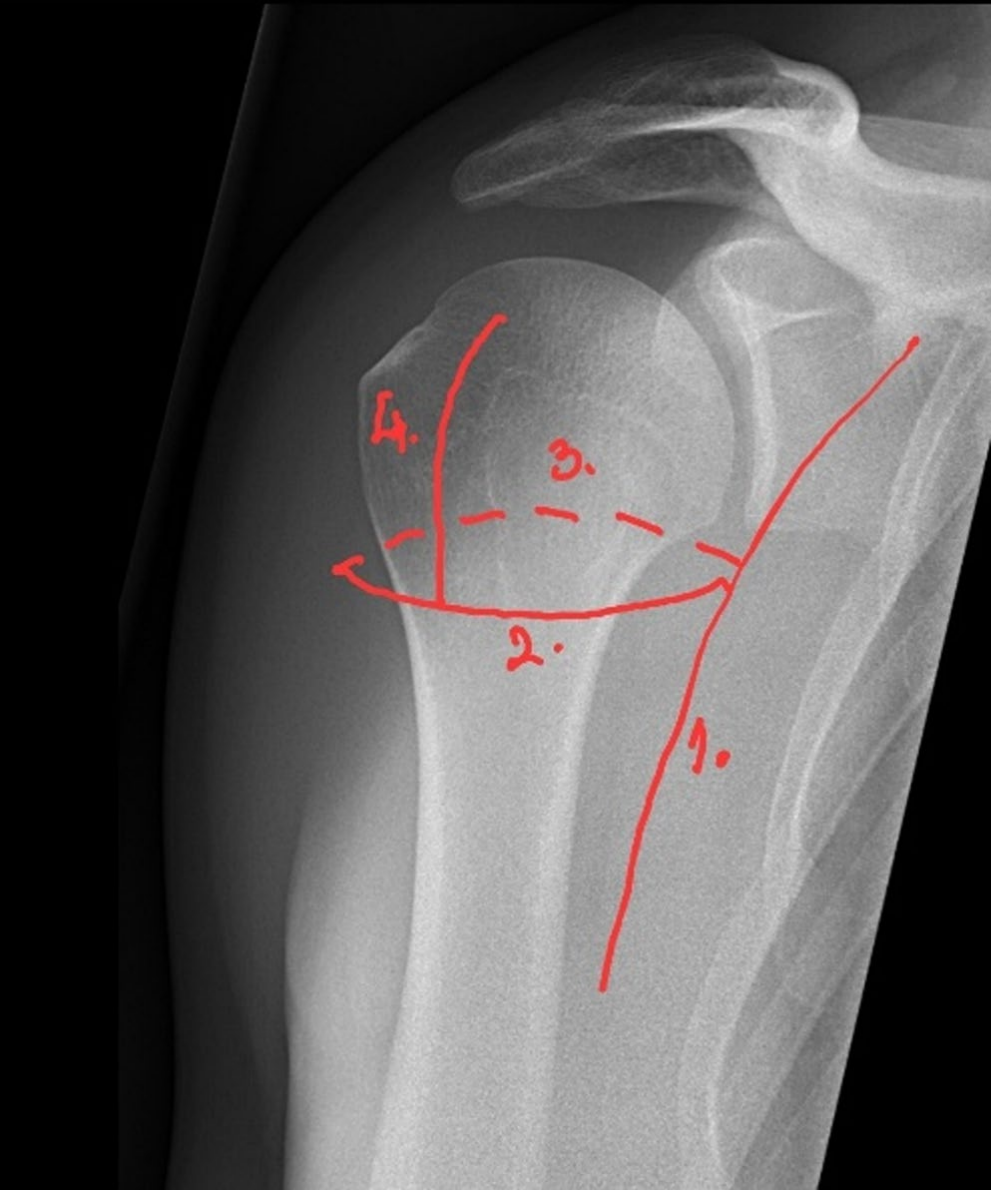
Blood Supply of the Proximal Humerus (1. Axillary Artery, 2. Anterior Circumflex Humeral Artery, 3. Posterior Circumflex Humeral Artery, 4. Arcuate Artery) [26]

The blood supply to the proximal humerus originates from branches of the axillary artery. Stronger and dominant posterior circumflex artery and the anterior circumflex artery, whose terminal branch (arcuate artery) is crucial for supplying the humeral head. Anastomoses between the circumflex arteries occur on the anterolateral aspect of the humerus. Additional vascular contributions arise from vessels associated with the rotator cuff [6, 7] (Fig. 2).

Stability and mobility of the joint primarily depend on the rotator cuff muscles (supraspinatus, infraspinatus, teres minor, subscapularis), deltoid, and pectoralis major. These muscles are innervated by branches of the brachial plexus, including the superior and inferior subscapular nerves, suprascapular nerve, axillary nerve, and lateral/medial pectoral nerves [8]. The axillary nerve is particularly vulnerable to injury during proximal humerus fractures and surgical procedures. After branching to the teres minor, it courses anteriorly along the undersurface of the deltoid at 2–7 cm distal to the acromion [9, 10].

Movement and stability at the glenohumeral joint rely on both static (bony anatomy, ligaments) and dynamic (muscles, tendons) stabilizers [11].

## Bone density

Various studies have investigated the distribution of bone tissue and bone density in the proximal humerus. Using different methods, they have shown that the impact of aging and changes in bone quality is a significant factor influencing the occurrence and type of fractures in the elderly.

The proximal part and medial side, especially near the articular region of the humeral head, exhibited the greatest amount of bone mineral density. The humeral neck had approximately half the bone mineral density of the humeral head. The cancellous bone of the neck had only one-third the mechanical strength of the humeral head. Trabecular bone has significantly higher density in the proximal posterior portion of the articular surface. The greater and lesser tuberosities and the central area of the proximal head had the lowest bone strength [12–17]. Wang and coworkers have found that the most prominent decrease in bone density due to age was observed in the region of the greater tuberosity, which is most pronounced in women during menopause [18].

According to Majeed the bicipital groove shows greater cortical bone thickness compared to the tuberosities [19].

Research results indicate that for every elderly individual being treated for a fracture of the proximal humerus, it is necessary to determine the quality of bone tissue. These findings must be considered during the planning of the procedure, the selection of implants, the angle of screw application, as well as the need for additional augmentation or corticocancellous grafting to ensure proper stability of the lateral implant.

## Osteoporosis

Fractures due to osteoporosis are at epidemic proportions worldwide. The number of elderly people with osteoporosis will increase by 32% in the USA between 2010 and 2030 [20]. Bone rigidity helps it resist external deformation and is made up of an inorganic mineral component, which is brittle but resists compressive forces, and an organic component, collagen, which gives the bone its elasticity. Bone strength is described as the sum of the mineral component of the bone (bone brittleness) and the collagen (elasticity) and resists an external force leaning on it and deforming it [21]. Bone strength is a joint function of bone mineral density, bone turnover, remodelling activity and the microarchitectural arrangement of the bone matrix, but it is also a property of the “material” (mineralisation rate), the denaturation of collagen, and the ability to repair microfractures in the matrix (trabecular microcracks). Bone strength therefore combines bone quantity, measurable by densitometry (DXA), and bone quality, measurable histomorphometrically, by micro-QCT, by some ultrasound experimental methods, and so on [22].

Nevitt’s coefficient of bone stiffness has the load on the bone from an external force (a fall from standing height, gravity) in the numerator and bone stiffness in the denominator. Fractures due to osteoporosis are therefore understood as both an accident and a disease since the fraction of the Nevitt coefficient of bone strength explains the fracture as an accident (a fall from standing height) in the numerator and as a disease (deformation of the bone due to reduced bone strength) in the denominator [22]. Treatment of the fracture must go parallel with the treatment of basic disease [23].

When planning treatment, it is necessary to be aware of the frequency of osteoporosis and reduced bone density in proximal humeral fractures. A simple solution that provides additional insight into bone condition is offered by Spross and coworkers in their study. They defined the deltoid tuberosity index (DTI), a simple one-level measurement on the AP fracture radiograph, and they found a very good correlation of DTI values of less than 1.4 with low bone quality of the humeral head measured on pQCT. The index is calculated just proximal to the deltoid tuberosity, where the outer cortical borders become parallel for the first time. At this level, the outer cortical diameter is divided by the inner endosteal diameter, resulting in a ratio that does not need to be corrected for the magnification error. The deltoid tuberosity index (DTI) has emerged as a reliable, simple radiographic tool to assess local bone quality in the proximal humerus. In validation studies, DTI has shown strong correlation with bone mineral density measured by peripheral quantitative computed tomography (pQCT). A DTI value below 1.4 indicates low bone mineral density with a sensitivity of 0.88 and specificity of 0.80. This measurement offers significant clinical advantages over other methods such as the Tingart measurement, particularly when fracture lines obscure traditional landmarks [24].

## Mechanism of injury

In the elderly population, fractures are caused by low energy, most frequently occurring at home with approximately 90% resulting from a fall from standing height [25]. A significant percentage suffer other injuries, such as hip fractures, which are the most common among fractures [26]. Nerve injuries can also occur, with a third of these resulting from a fall from standing height. The most injured nerves are the axillary nerve and the suprascapular nerve [27]. Despite the low energy, significant dislocations can lead to injury of the axillary artery. Even with the presence of a pulse, attention must be paid to more subtle signs of ischemia, and additional imaging diagnostics should be performed without delay [28].

The shoulder joint has the greatest mobility in the entire body, allowing for countless positions of the upper extremity during injury. Therefore, it is very difficult to precisely determine the mechanism of injury that led to the fracture [8]. In addition to fractures, pure dislocations (anterior, inferior, posterior) are also possible, as well as dislocations with fractures.

The type of fracture depends on the kinetic energy, the position of the upper extremity during injury, and the quality of the bone [28]. Proximal humeral fractures occur with the spread of the fracture line through the epiphyseal lines, as initially described by Codman, with four separate fragments. These include the head, the greater tuberosity, the lesser tuberosity, and the diaphysis [29].

The mechanism is direct, from a blow or fall onto the shoulder. Contact occurs between the harder bone of the glenoid, which acts as an anvil, and the softer proximal humerus, causing a humeral fracture. These are usually higher energy and often result in comminute fractures with the head in a varus position, which due to natural retroversion is tuned inferiorly and posteriorly. However, an isolated fracture of the greater tuberosity or surgical neck can also occur [30]. In the elderly, the mechanism is usually indirect, from a fall on an outstretched arm in various arm positions, which mostly leads to a fracture with the head in a valgus position. In cases of pronounced external rotation, an isolated fracture of the greater tuberosity may occur [31]. Edelson with coworkers describes the “parachute mechanism” as a protective, reflexive movement during a fall on an outstretched arm at 90° abduction, lateral or ventral, which causes three different types: an undisplaced fracture of the greater tuberosity, a “shield-type” in which the arcuate artery in the bicipital sulcus is preserved (i.e., blood supply to the head is maintained), and a split head or complex three- and four-part fractures [32, 33]. Isolated fractures of the lesser tuberosity are extremely rare [28].

Due to the lower bone density in certain areas of the proximal humerus, fracture lines most commonly appear in the region of the surgical neck, followed by the greater tuberosity, anatomical neck, and lesser tuberosity. Head split and fractures at the bicipital sulcus region are rare. Because the energy is usually low, 65% of the fractures in the elderly group are undisplaced, and most commonly result in two-part fractures of the surgical neck. These are followed by fractures of the greater tuberosity and then more complex three- and four-part fractures. Complex fractures occur in 21% of cases [34].

Regardless of the mechanism, good predictors of ischemia include the length of the metaphyseal extension of the head, preservation of the calcar and medial hinge, as well as a simpler fracture pattern. Poor predictors of ischemia include four-part fractures, angular dislocation, the degree of tuberosity dislocation, glenohumeral dislocation, head splitting, and three-part fractures [35].

Once a fracture occurs, additional dislocation can be caused by the tendons of muscles that attach to the proximal humerus. The supraspinatus, infraspinatus, and teres minor muscles pull the greater tuberosity cranially and posteriorly. The subscapularis muscle dislocates the lesser tuberosity medially. The deltoid, pectoralis major, latissimus dorsi, and teres major muscles dislocate the diaphysis anteriorly and somewhat cranially. Due to natural retroversion, in multi-part fractures, the head rotates posteriorly and inferiorly [8].

## Clinical examination

Initial care follows the Advanced Trauma Life Support (ATLS) protocol. After stabilizing vital functions, a detailed medical history and thorough clinical examination are conducted.

The mechanism of injury, prior shoulder injuries, and preexisting shoulder conditions are determined. The patient’s general health, comorbidities and treatment expectations are assessed. The injured shoulder is painful, with pain worsening during movement and palpation. The patient typically holds the arm in an antalgic position, pressed against the torso. The shoulder is swollen due to tissue bleeding, often with visible bruising extending downward along the limb and onto the chest. Deformity is evident in fractures with significant fragment displacement or dislocation. Active shoulder movement is impossible, and attempts at passive mobility cause severe pain and crepitus. Fracture stability is assessed by gently rotating the humerus while palpating the proximal portion. If the humerus moves as a unit, the fracture is stable.

The examination must identify potential nerve injuries, vascular impairments, or wounds related to the fracture. Axillary nerve injury may cause sensory disturbances over the deltoid muscle. Suprascapular nerve injury results in posterior shoulder pain and rotator cuff weakness, while brachial plexus injury (less common) causes limb weakness and numbness. Many nerve injuries are asymptomatic and are only detected via electromyography (EMG). In an EMG study, Visser documented a 67% incidence of nerve injuries in low-energy proximal humerus fractures, most commonly involving the axillary (58%), suprascapular (48%), radial (32%), and musculocutaneous (29%) nerves, particularly in displaced fractures [27]. Most neurological deficits are transient; permanent impairments are rare (< 5%).

Deltoid muscle atony may occur after proximal humerus fractures or surgery. This causes inferior subluxation of the humeral head, visible on anteroposterior (AP) radiographs. It is not due to axillary nerve injury and typically resolves spontaneously within weeks.

Axillary artery injury is rare (< 0.1%) but serious. It may result from fragment compression, stretching, or direct vascular trauma. Distal pulses may remain intact due to collateral circulation. Risk increases with associated injuries (brachial plexus, scapular fractures), open fractures, displaced fractures with medial humeral head displacement, and in elderly patients [36].

## Imaging

### Conventional radiography

Neer recommended using anteroposterior (AP) and scapular Y-view projections to assess the fracture pattern of the proximal humerus. Over the years, other authors have advocated for additional axillary projections, which best visualize tuberosity displacement and humeral head fractures or dislocations. These projections form the basic trauma series of radiographs for evaluating proximal humerus fractures. With this series, the glenohumeral joint and proximal humerus are assessed in three orthogonal planes [37].

For the **anteroposterior (AP) view**, the X-ray beam is angled 45 degrees to the sagittal plane (perpendicular to the scapular axis). This projection clearly visualizes the articular surface of the humeral head and glenoid cavity and is also suitable for evaluating the tuberosities.

**Fig. 3:**
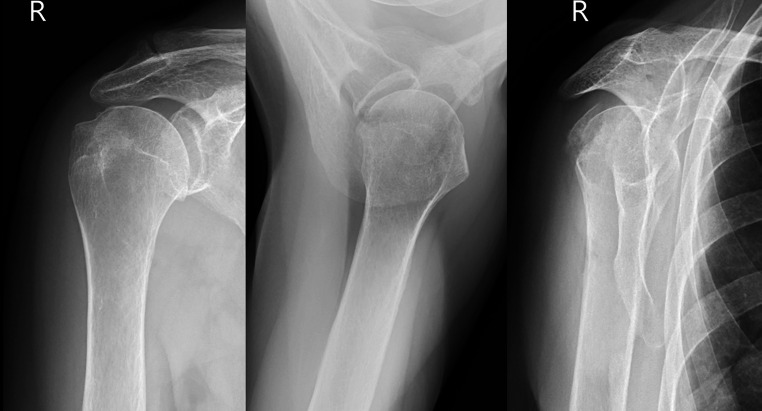
Basic trauma series of radiographs for evaluating a right proximal humerus fracture of 76 years old female with fracture of the greater tubercle adequately visible only on scapular Y view [37]

For the **axillary view**, the beam is directed toward the patient’s head with the arm abducted (70–90 degrees), and the cassette is placed at the top of the shoulder. This projection is optimal for assessing dislocations, humeral head fractures, glenoid involvement, and lesser tuberosity fractures. If the patient cannot abduct the arm due to pain, a **Velpeau axillary view** is performed. The patient remains in a shoulder brace and leans back 30–45 degrees over the cassette. The beam is then directed downward, perpendicular to the cassette. For the **scapular Y-view**, the beam is angled 40 degrees from the coronal plane in a PA direction (perpendicular to the standard AP view). This projection displays the scapula in a Y-shape: the lower limb of the Y represents the scapular body, while the upper limbs form the coracoid process and scapular spine with the acromion. The glenoid is seen at the centre of the Y, and the humeral head aligns with the intersection of the Y’s limbs (Fig. 3) [37].

**Fig. 4:**
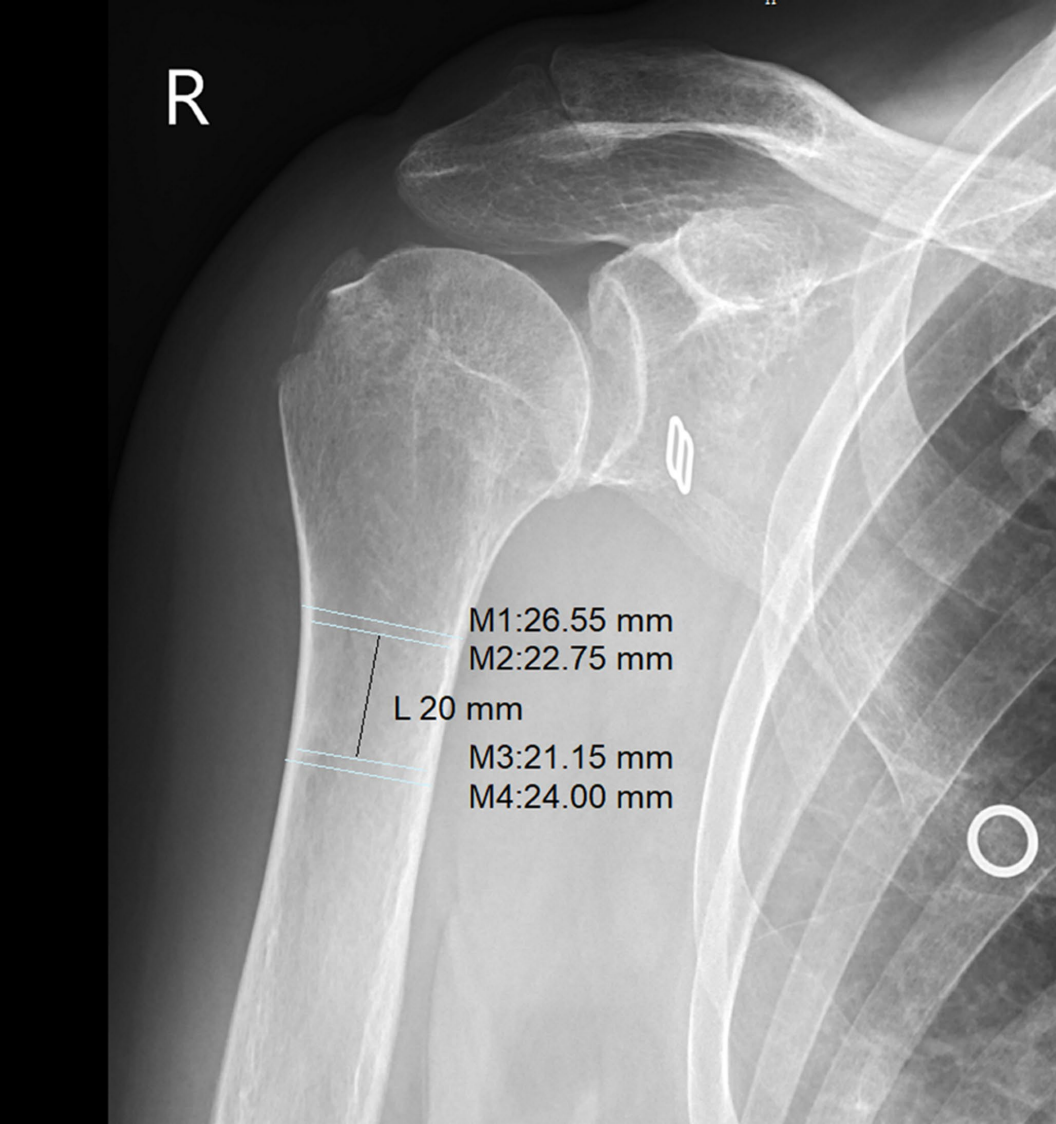
Cortical thickness measurement in a 76-year-old female with a fracture of the greater tubercle, as defined by Tingart [16]. The combined cortical thickness (CCT), calculated as [(M2 − M1) + (M4 − M3)]/2, measured < 4 mm, suggesting osteoporosis. Her bone mineral density (BMD) measurement in the distal forearm revealed a T-score of − 2.8

**Bone quality** is a critical factor in decision-making for proximal humerus fractures. Bone mineral density (BMD) can be estimated radiographically. Tingart compared the cortical thickness of the proximal humeral diaphysis with the BMD of the proximal humerus. He found that a cortical thickness of less than 4 mm is a strong indicator of low BMD [16] (Fig. 4).

Adequate **vascularization of the humeral head** is crucial for fracture healing. Avascular necrosis (AVN) and impaired fracture healing are significant complications, especially in fractures involving the anatomical neck and displaced four-part fractures. Fractures involving the anatomical neck, four-part fractures, significant fragment displacement, varus malalignment, bone defects in the calcar region, and fractures with dislocation visible on radiographs are potential predictors of compromised vascularization. Hertel demonstrated that fractures of the anatomical neck with a short metaphyseal segment (< 8 mm) and disruption of the postero-medial periosteal hinge have a 97% predictive value for humeral head ischemia [35].

### CT scan

**Computed Tomography (CT)** is a key imaging modality for evaluating proximal humerus fractures, particularly in complex cases where radiographs are insufficient. It is indicated for complex 3- and 4-part fractures, fractures with humeral head split, dislocations, and glenoid involvement [4, 38].

The reproducibility of fracture classification systems like Neer and AO/OTA for proximal humerus fractures has historically been limited due to challenges in accurately visualizing complex fracture patterns on 2D radiographs. Brunner et al. (2009) demonstrated that **3D CT reconstruction** eliminates overlapping structures, enabling surgeons to significantly improve classification accuracy and interobserver agreement. It assesses tuberosity displacement (greater/lesser) and humeral head integrity (split vs. intact). It measures displacement/angulation objectively (e.g., medial calcar disruption, varus/valgus alignment). It allows visualisation of critical anatomic landmarks like medial calcar integrity where cortical bone loss is a key predictor of instability and fixation failure and posteromedial hinge where disruption of this hinge (a Hertel criterion) correlates with humeral head ischemia (AVN risk) [39].

**Fig. 5:**
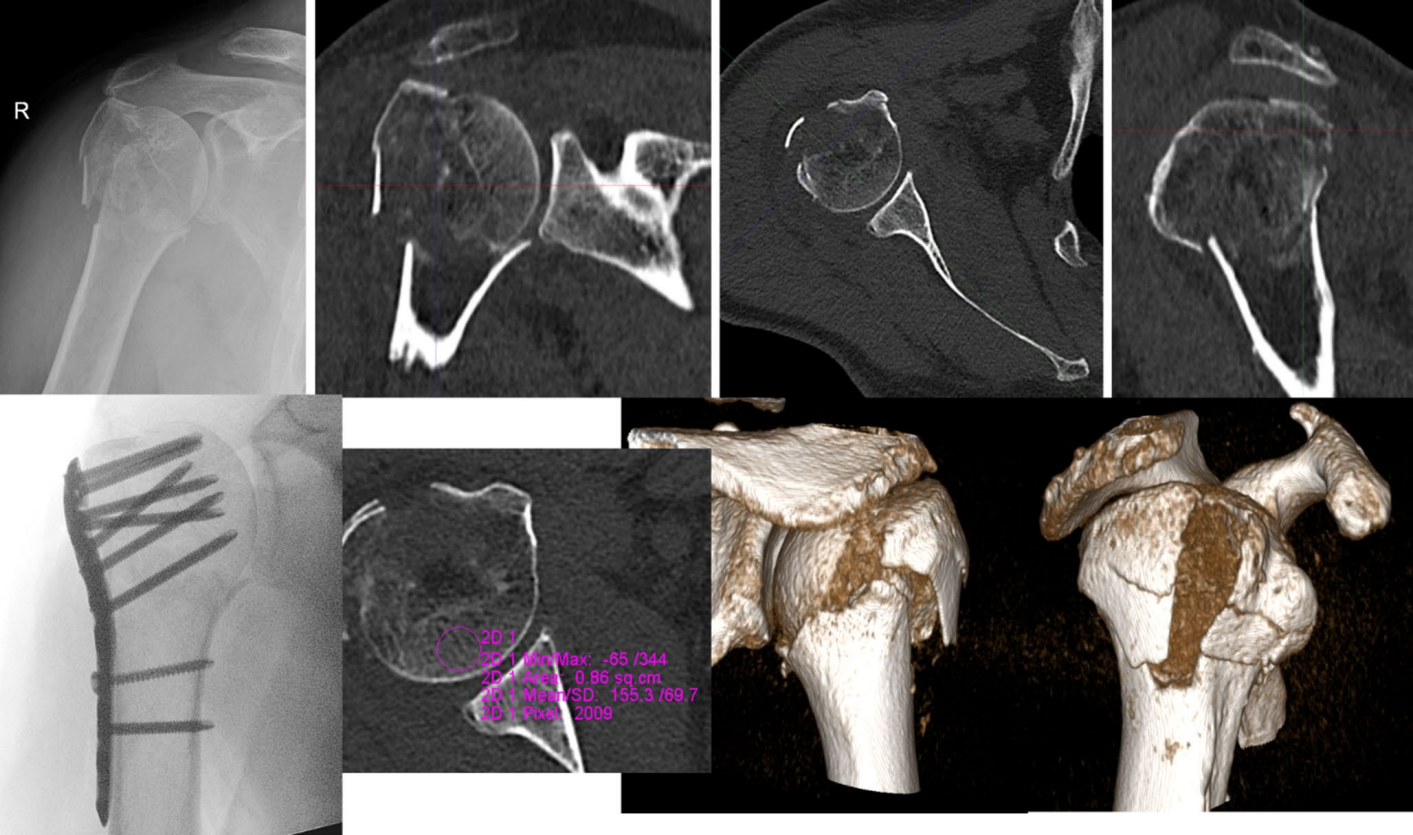
Computed tomography (CT) scan of a four-part proximal humerus fracture in a 66-year-old male with good bone quality (Hounsfield units [HU] > 100), managed with osteosynthesis [40]

CT enables **quantitative and qualitative bone structure analysis**. CT images can measure bone density in Hounsfield Units (HU). Measurement sites are trabecular bone in the centre of the humeral head (critical for screw purchase in fixation), greater tuberosity (assesses bone quality for suture anchor fixation) and calcar region (predict medial column stability). A threshold of < 100 HU is often associated with poor bone quality and higher fracture risk [40]. Low HU values suggest increased risk of fixation failure (e.g., screw cut-out, implant loosening). In patients with severe osteoporosis, conservative treatment or reverse shoulder arthroplasty may be preferred over internal fixation. CT also visualizes trabecular patterns and areas of poor bone quality. Thin or disrupted cortical bone in the calcar region is critical for assessing fixation stability (Fig. 5).

**Advanced CT techniques** for bone evaluation include Dual-Energy CT (DECT) and 3D volumetric analysis. **DECT** uses two distinct X-ray energy spectra to differentiate materials based on their atomic composition. This removes calcium signals from bone, highlighting bone marrow edema, occult fractures, or soft tissue injuries. It detects hidden fractures in the humeral head (e.g., “head-split” fractures) or bone bruising that may predict avascular necrosis (AVN) risk. It measures BMD more accurately than single-energy CT by analyzing material decomposition (e.g., calcium vs. fat) [41–44]. There is less need for additional DEXA and MRI.

### MRI

While MRI is excellent diagnostic tool, most proximal humerus fractures can be managed with X-rays and CT. MRI shines in scenarios where traditional imaging falls short. For instance, elderly patients with persistent shoulder pain and normal X-rays may harbour occult fractures—small cracks in the humeral head or greater tuberosity invisible to other modalities. MRI is equally vital for evaluating soft tissue injuries. Up to 30% of proximal humerus fractures involve concurrent rotator cuff tears, which, if missed, can lead to poor outcomes. MRI visualizes tendon integrity in detail, distinguishing partial tears from full-thickness ruptures. Similarly, labral tears or biceps tendon injuries—common in fractures with shoulder dislocation—are clearly depicted, guiding decisions about surgical repair [45, 46]. For complex fractures, it also evaluates the vascular risk to the humeral head, a critical factor in predicting AVN. Early signs of ischemia, such as bone marrow edema or disrupted blood supply, appear on MRI long before X-rays show collapse or sclerosis [47]. Despite its strengths, MRI has limitations. It’s less accessible than CT, costlier, and contraindicated in patients with pacemakers or metallic implants. Metal artifacts from prior hardware can also obscure images. Additionally, MRI cannot match CT’s precision in visualizing complex fracture lines or measuring displacement.

### Classifications of proximal humerus fractures

Fracture classification is crucial as it guides clinicians in treatment decisions [48]. It arises from the need to understand fracture morphology, make decisions about the treatment approach, and monitor treatment outcomes. Over time, various classifications have been proposed, but none has succeeded in achieving all the outlined goals. The ideal classification should be based on a unified diagnostic algorithm, reliably guiding the treatment method and implant selection while accurately predicting the final treatment outcome. At the same time, it should be simple to use, regardless of the clinician’s experience.

One of the earliest attempts at classification was made by Ernest Codman in 1934. Codman’s system divided the proximal humerus into four main anatomical parts: the humeral shaft, the articular surface (humeral head), the greater tuberosity, and the lesser tuberosity. He identified 16 possible combinations Codman’s classification was limited by its lack of consideration for fracture displacement and angulation, which are critical factors in determining treatment and prognosis [49].

**Fig. 6:**
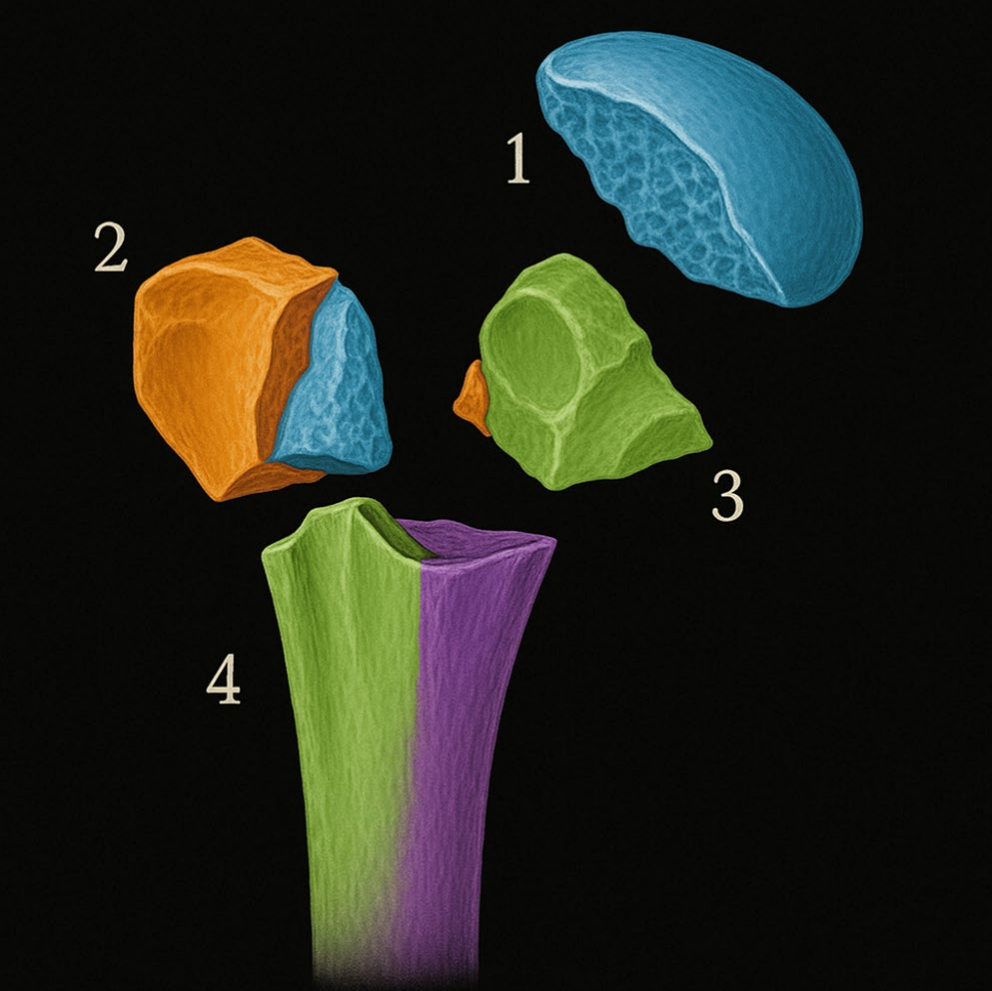
Segments of The Proximal Humerus according to Codman’s and Neer’s Classification, Adopted from: **Neer CS 2nd**. Displaced proximal humeral fractures. I. Classification and evaluation. *J Bone Joint Surg Am* [50]

In 1970, Charles Neer introduced a classification system that built upon Codman’s work by incorporating the concept of displacement. He set an arbitrary threshold of 1 cm or 45 degrees for displaced fragments and originally describes six groups. Neer later revised his classification, focusing on four fragments and abandoning the six groups. A one-part fractures comprises all undislocated fractures, regardless number of fracture lines. Two-part fracture involves the displacement of one of the four segments. For example, surgical neck (SN) displacement fracture with displaced head. Another example is a greater tuberosity fracture associated with anterior shoulder dislocation. Three-part fracture means two of the four segments are displaced. A common pattern is displaced SN plus one of tuberosities, while the remaining tuberosity remains attached to the humeral head. And four-part fractures are the most severe, characterized by the displacement of all four segments. In these injuries, the humeral head is typically displaced and loses contact with the glenoid. Four-part fractures carry a high risk of avascular necrosis (AVN) of the humeral head due to disruption of its blood supply. Neer later added valgus-impacted four-part fractures as a distinct category in 2002. Neer’s classification includes separate categories for fracture-dislocations and articular surface injuries such as head-splitting fractures and impaction fractures [50]. Neer’s system remains one of the most widely used classifications for proximal humerus fractures [49] (Fig. 6).

**Fig. 7:**
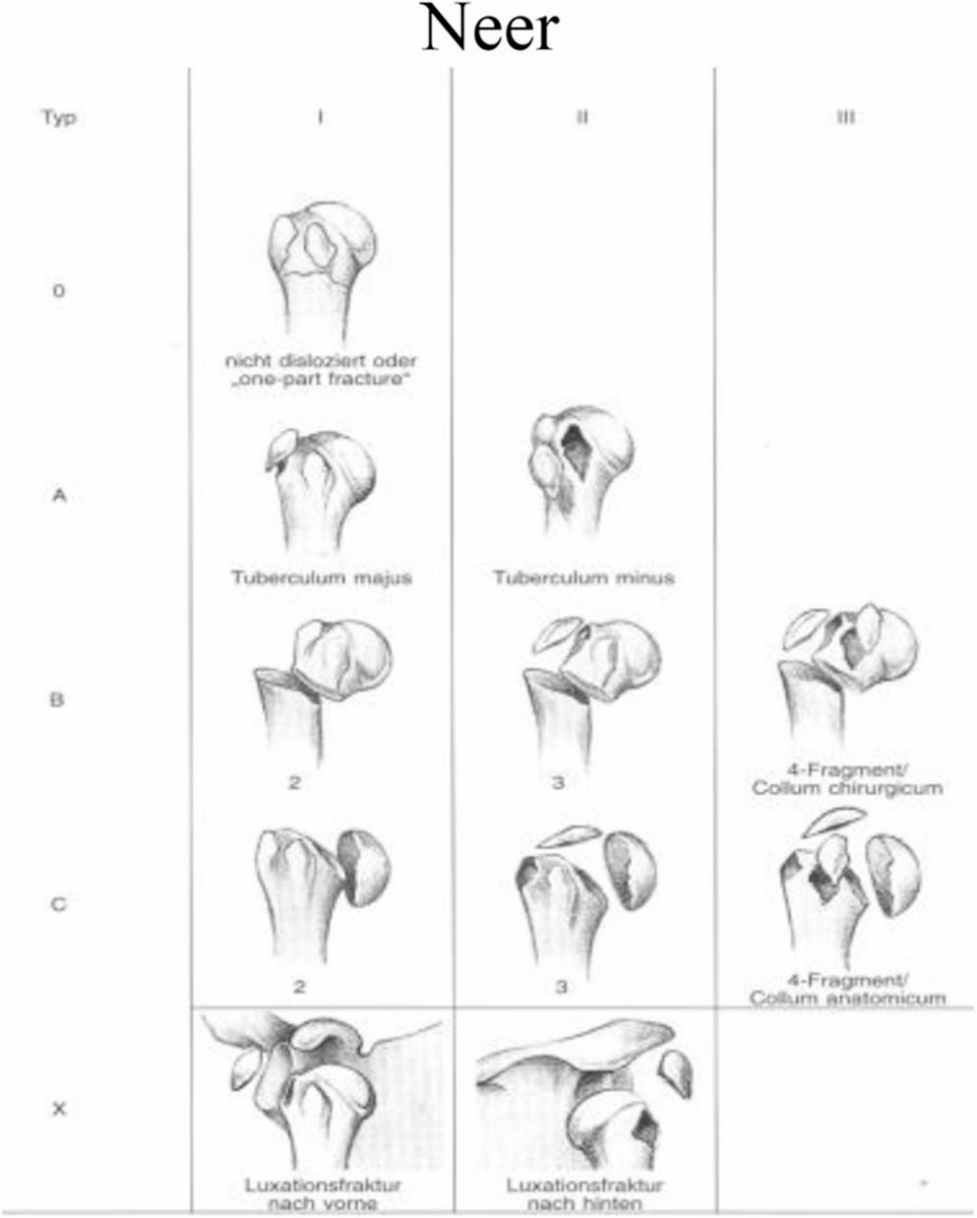
Neer CS 2nd. Displaced proximal humeral fractures. *J Bone Joint Surg* [51]

Although Neer included the four-part valgus impacted fracture in his classification, its true significance was highlighted by Jakob and colleagues. In this specific pattern, the humeral head is driven into a valgus position between the tuberosities, which splay out. Unlike the classic four-part fracture, the articular surface maintains contact with the glenoid, and this variant often has a different prognosis and treatment approach. AVN is only described in 26% of cases [51] (Fig. 7).

**Fig. 8:**
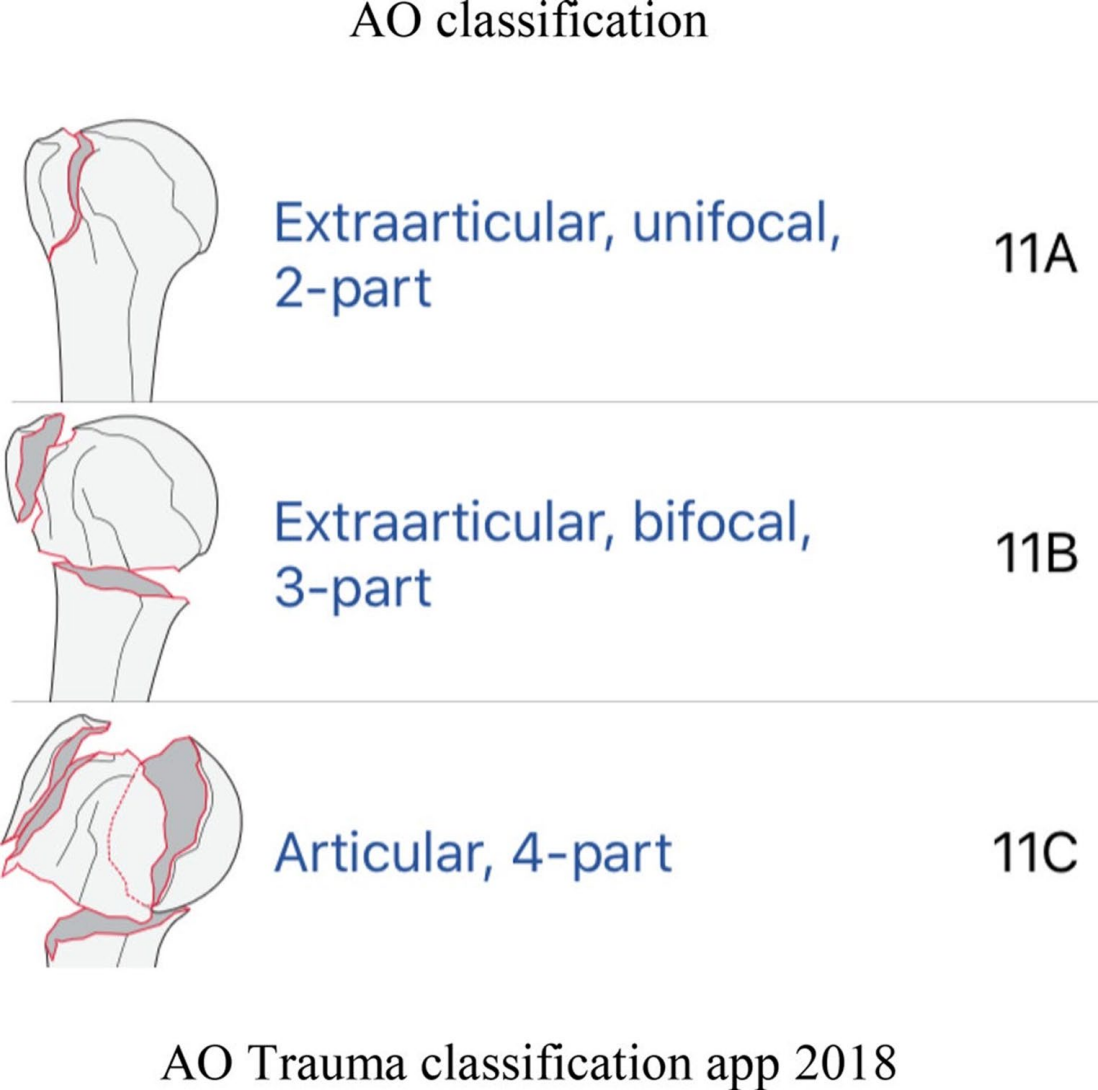
Müller ME. The Comprehensive Classification of Fractures of Long Bones [52]

Following Neer, the Arbeitsgemeinschaft für Osteosynthesefragen/Orthopaedic Trauma Association (AO/OTA) system was developed in the 1980 s and later revised in 2018 (Fig.8) [52]. This classification offers a more detailed approach, categorizing fractures into three main types (A, B, and C), which are further subdivided into groups and subgroups based on increasing complexity and prognostic implications. Due to the complexity of this classification, its daily use is very complicated, and as a result, it has not been widely adopted by clinicians. Due to the issues, the classification was revised and simplified. Modifiers were introduced to provide additional information on associated injuries, such as soft tissue, vascular, and nerve injuries, as well as dislocations and fragment shifts.

**Fig. 9:**
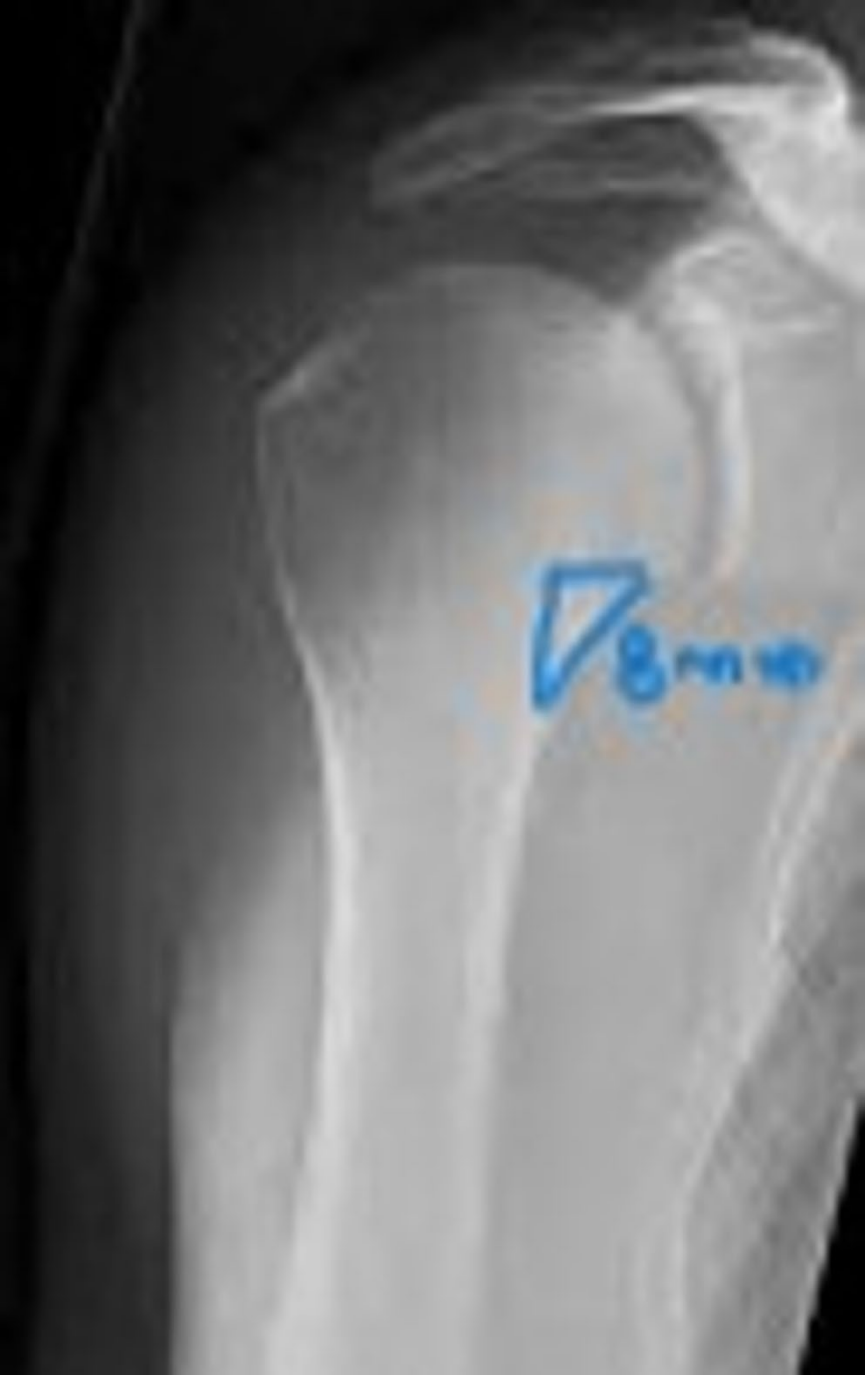
Calcar length according to Hertel predicts incidence of AVN [35]

The Codman-Hertel system, also known as Codman-Hertel or Hertel’s Lego classification, emerged as another significant contribution, and offers a unique binary approach to describing proximal humerus fractures. Instead of focusing primarily on the number of displaced segments, this system is based on the presence or absence of fractures in five basic fracture planes [35]. For a complete description of the fracture, five key questions need to be answered with “yes” or “no.” The questions are: (1) Is there a fracture line between the head and the greater tuberosity? (2) Is there a fracture line between the greater tuberosity and the diaphysis? (3) Is there a fracture line between the head and the lesser tuberosity? (4) Is there a fracture line between the lesser tuberosity and the diaphysis? Finally, (5) Is there a fracture line between the greater and lesser tuberosity [53]? A significant advantage of the Hertel classification is its incorporation of factors that are strong predictors of humeral head ischemia, such as the length of the medial metaphyseal extension (calcar length) and the integrity of the medial hinge. These factors are crucial for assessing the risk of avascular necrosis (AVN) (Fig. 9).

**Fig. 10:**
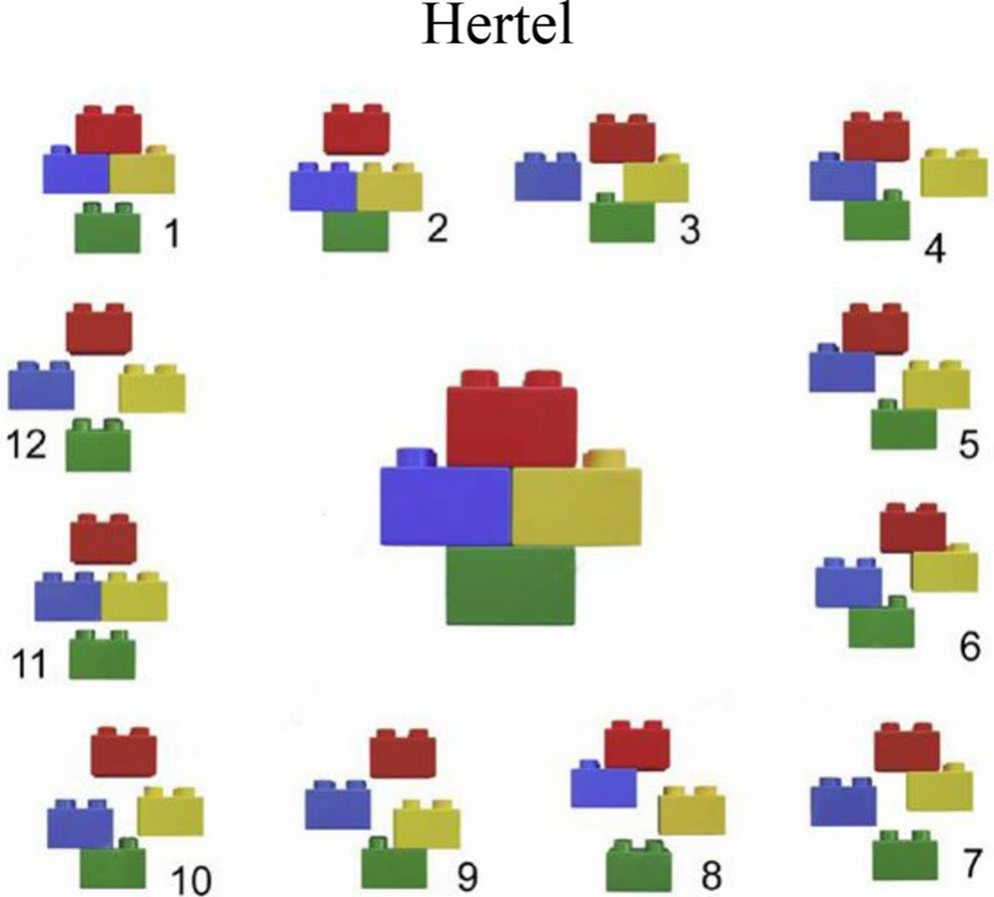
**Hertel R**, Hempfing A, Stiehler M, Leunig M. Predictors of humeral head ischemia after intracapsular fracture of the proximal humerus. J Shoulder Elbow Surg. Am [35]

The system also uses a nomenclature based on the parts of the proximal humerus: Head (H), Greater Tuberosity (G), Lesser Tuberosity (L), and Shaft (S). Fracture planes are denoted by a hyphen, allowing for a descriptive representation of the fracture pattern (e.g., H-GLS for an anatomical neck fracture, H-G-L-S for a four-part fracture). All fracture types could be systemized in one of 12 + 2 groups [35] (Fig. 10).

More recently, the Edelson classification was proposed as a three-dimensional system utilizing computed tomography (CT) and 3D reconstructions to visualize the complex fracture geometry, fracture patterns and their relationship to injury mechanisms [32, 54]. The research by Edelson and Armitage has laid the foundation for a new era of studies that require the use of more advanced technologies and powerful diagnostic tools, enabling fracture mapping [54, 55]. This technique provides a visual representation of fracture morphology by overlaying fracture lines from the model onto the normal model using 3D CT diagnostics. This method allows for a complete visual illustration of the fracture’s position, direction of spread, and division [14, 22, 56–58]. It also considers bone density, suggesting that in the future, the use of this technology could provide a reliable classification of fractures in elderly patients. Examples of such studies include those by Liu and colleagues, and Mys and colleagues, who, through mapping, confirmed the complexity of four-part fractures [59, 60]. They highlighted the frequency of comminution in the anatomical neck area and, unlike previous studies, found that fractures involving the bicipital sulcus were more common than previously thought, while those involving the articular surface were somewhat rarer.

**Fig. 11:**
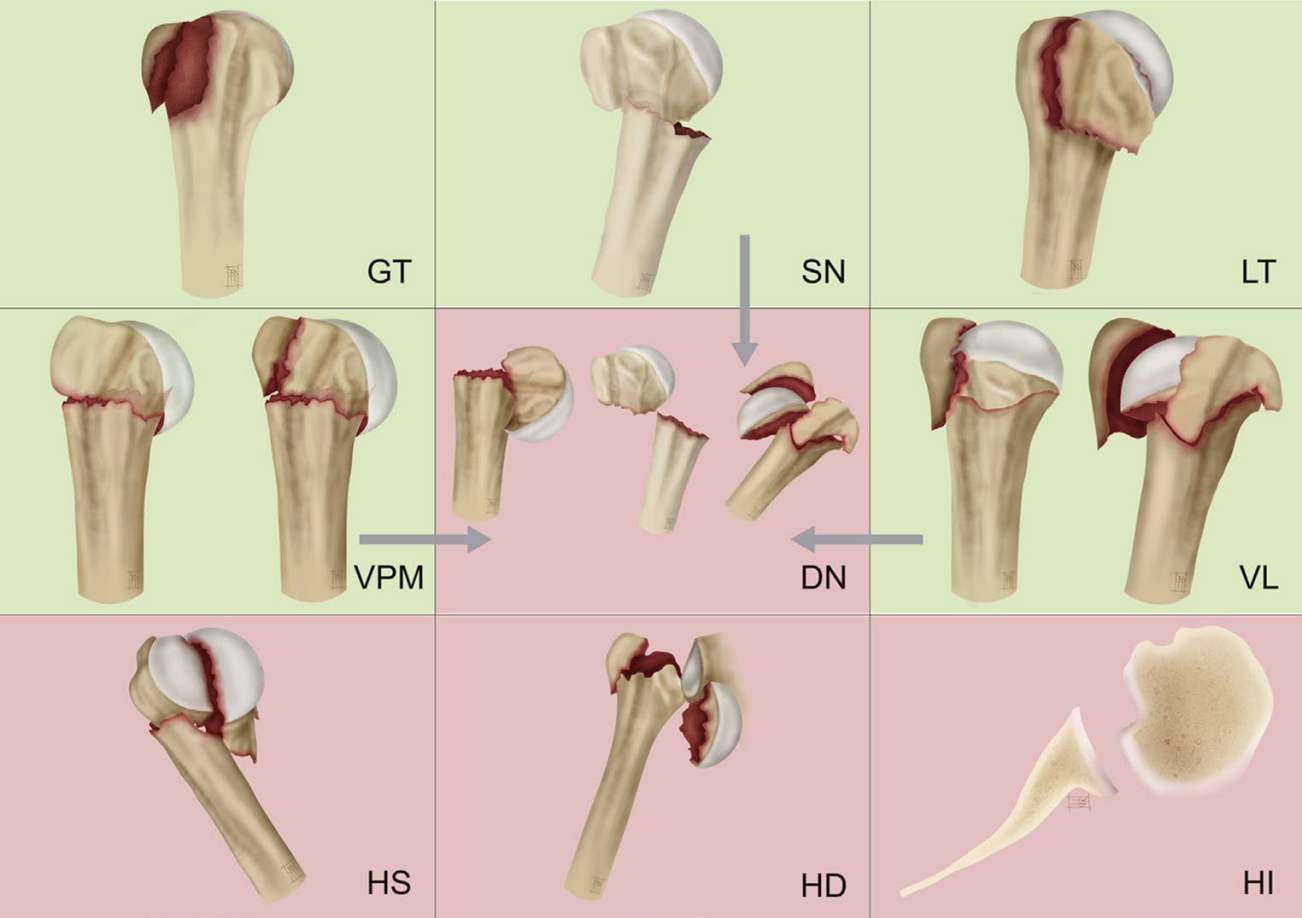
Adopted from: **Foruria AM**, Martinez-Catalan N, Pardos B, et al. Classification of proximal humerus fractures according to pattern recognition is associated with high intraobserver and interobserver agreement. *JSES Int*. 2022;6:563–568 [61]

A very interesting and useful classification was developed by Foruria and colleagues, called the Mayo/Fundacion Jimenez Diaz (Mayo/FDJ) Classification, is based on X-ray and multiplanar CT with 3D reconstruction. It identifies 4 fracture planes and 7 patterns. The most common fracture planes are the surgical neck fracture plane, the greater tuberosity fracture plane, the lesser tuberosity, and the anatomic neck fracture plane. The fracture patterns include surgical neck fractures, isolated tuberosity fractures, varus posteromedial (VPM) impaction fractures, valgus impacted (VI) fractures, disengaged neck fractures, fracture dislocations, head splitting, and head impaction [61]. The classification has proven reliable in correlating fracture patterns and displacement with the outcomes of non-operatively treated fractures [62]. It offers a framework to assist in making treatment decisions [63] (Fig. 11).

### Clinical significance and comparison of classification systems

The various classification systems for proximal humerus fractures play a critical role in guiding clinical decision-making. The choice of treatment, whether non-operative or surgical, is often influenced by the specific fracture pattern as defined by these systems. For instance, minimally displaced one-part fractures, according to Neer’s classification, are typically managed non-operatively with immobilization in a sling and early range of motion exercises. Conversely, displaced two-, three-, and four-part fractures, as well as fracture-dislocations, often require surgical intervention, which may include open reduction and internal fixation (ORIF) with plates and screws, hemiarthroplasty (replacement of the humeral head), or reverse total shoulder arthroplasty, particularly in elderly patients with poor bone quality or rotator cuff deficiencies.

The inter-observer reliability of these classification systems is an important factor in their clinical utility. Studies have shown varying degrees of reliability among the different systems. While the Neer and AO/OTA classifications are widely used, they have been reported to have moderate to low inter-observer reliability in some studies, potentially due to the subjective nature of assessing displacement and the complexity of the AO/OTA system [33, 64]. A particular challenge has been the precise determination of 1 cm displacement on X-rays, which are not standardized and vary significantly in quality, directly impacting reproducibility [65, 66].

In contrast, the Hertel classification has shown higher inter-observer reliability in certain studies, possibly due to its more objective binary approach based on fracture planes.

The revised Edelson classification, with its three-dimensional assessment, has also demonstrated satisfactory to excellent reliability.

The Mayo FDJ classification has proven reliable in correlating fracture patterns and displacement with the outcomes of non-operatively treated fractures. It offers a framework to assist in making treatment decisions. A recent study confirmed consensus, with adequate intra- and interobserver reliability using both X-ray and CT scans [61, 62].

## Conclusion

The classification of proximal humerus fractures is a critical aspect of orthopaedic trauma practice, influencing diagnosis, treatment planning, and prognosis. Even though classification systems provide valuable guidance, treatment decisions should also consider the patient’s age, comorbidity, activity level, bone quality, and the presence of any associated injuries. Continued research and evaluation of these systems are crucial for further improving their reliability, validity, and clinical utility, ultimately leading to better outcomes for patients with proximal humerus fractures [67].

## Data Availability

No datasets were generated or analysed during the current study.
